# Supplementary material for: The causal role of circulating inflammatory markers in osteoporosis: a bidirectional Mendelian randomized study
Source: Front Immunol. 2024 Jul 18;15:1412298. doi: 10.3389/fimmu.2024.1412298 (PMC11291241; doi:10.3389/fimmu.2024.1412298)
Supplement: Supplementary file 1 [file DataSheet_1.zip › supplementary material/CIM-OSTEOPOROSIS /GCST90274841.csv_forest.pdf]

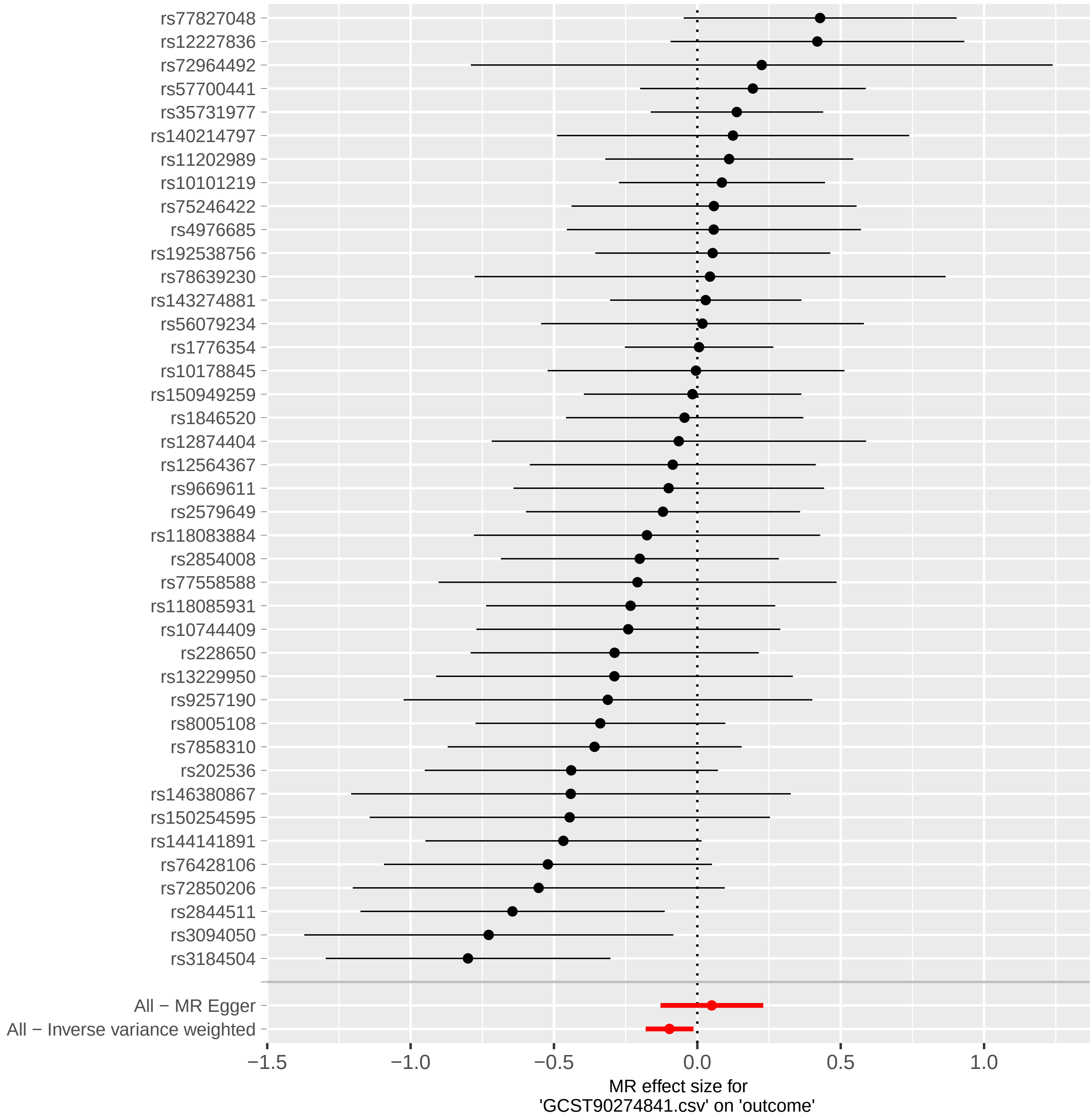
rs77827048
rs12227836
rs72964492
rs57700441
rs35731977
rs140214797
rs11202989
rs10101219
rs75246422
rs4976685
rs192538756
rs78639230
rs143274881
rs56079234
rs1776354
rs10178845
rs150949259
rs1846520
rs12874404
rs12564367
rs9669611
rs2579649
rs118083884
rs2854008
rs77558588
rs118085931
rs10744409
rs228650
rs13229950
rs9257190
rs8005108
rs7858310
rs202536
rs146380867
rs150254595
rs144141891
rs76428106
rs72850206
rs2844511
rs3094050
rs3184504
All − MR Egger
All − Inverse variance weighted
−1.5
−1.0
−0.5
0.0
0.5
1.0
MR effect size for
'GCST90274841.csv' on 'outcome'
